# Cardiovascular risk knowledge in patients of South Asian origin living with rheumatoid arthritis: data from India and the UK

**DOI:** 10.1186/s41927-020-00156-1

**Published:** 2020-10-20

**Authors:** Kanta Kumar, Suvrat Arya, Peter Nightingale, Tom Sheeran, Amita Aggarwal

**Affiliations:** 1 Institute of Clinical Sciences, College of Medical and Dental Sciences, University of Birmingham, Birmingham, B15 2TT UK; 2 Department of Clinical Immunology and Rheumatology, Sanjay Gandhi postgraduate Institute of Medical Sciences, Lucknow, India; 3 Institute of Translational Medicine, University Hospitals Birmingham NHS Foundation Trust, Birmingham, B15, 2TH UK; 4 Department of Rheumatology, Royal Wolverhampton NHS Trust, Wolverhampton, WV10 0QP UK

**Keywords:** Rheumatoid arthritis, Cardiovascular risk, Ethnicity, Knowledge, Cross sectional survey

## Abstract

**Background:**

South Asians have a higher risk of cardiovascular disease (CVD). Rheumatoid arthritis (RA) increases the risk of premature atherosclerosis. We investigated whether there was a substantial difference in the level of CVD risk knowledge among patients of South Asian origin with RA in India and in the UK.

**Methods:**

In this cross-sectional survey, patients of South Asian origin with RA from India and the UK were recruited from secondary care settings. Data were collected via Heart Disease Fact Questionnaire-Rheumatoid Arthritis (HDFQ-RA), a validated self-completion questionnaire. The HDFQ-RA was translated into Hindi and piloted among patients from South Asian background before use. Additionally, clinical and demographic data was collected.

**Results:**

Among 118 patients from each country, 84% were female and they had similar age, education level, employment status and co-morbidities. Patients from India had longer disease duration (5.5 years versus 4.1 years (*p* = 0.012) whereas those from the UK had higher disease activity score (4.0 + 0.8 versus 3.1 + 0.7, *p* < 0.01). Regarding modifiable risk factors for CVD only 51.2% from India and 51.3% in the UK were aware of them. However, awareness of the link between RA and increased risk of CVD was even more limited (32.8% in India and 34.4% in UK).

**Conclusion:**

Patients of South Asians origin with RA from both countries had limited knowledge about CVD risk. There is a need to educate them about CVD risk during consultation, as this will result in better outcomes.

## Background

Cardiovascular diseases (CVD) related to atherosclerosis; include ischaemic heart disease, stroke and peripheral vascular disease [1]. CVD is the leading causes of death in most of high-low- and middle-income countries [2]. Low and middle-income countries account for more than 80% of global deaths and 85% of global disability from CVD [2, 3]. CVD risk is known to escalate in certain co-morbid diseases such as rheumatoid arthritis (RA) [4]. Patients with RA have increased rates of CVD for reasons that are not fully understood [5]. Contributing factors include persistent inflammation related to systemic disease activity, use of medications such as glucocorticoids and possibly the effects of cytokines [6]. The overall increased risk of CVD in the RA population exceeds that predicted by increased rates of traditional risk factors [1].

Globally, it has been noted that controlling comorbidities such as hypertension and signs of dyslipidaemia very much fall into the domain of a rheumatologist [1]. As a result, European League Against Rheumatism (EULAR) present clear recommendations on managing CVD risk in RA patients [1]. The consequences of accelerated atherosclerosis include an increased risk of cerebrovascular and coronary heart disease (CHD) [7]. This is particularly so in South Asian population where the effect of RA itself on premature CVD appears to be substantial [8]. In addition, the South Asian population has a baseline higher prevalence of CVD [9]. In RA, about a third of patients of South Asian origin with active disease have higher levels of subclinical atherosclerosis than other RA population [8]. These data mainly emerge from India and are limited in other parts of the European countries where migrated population have settled over time. Patient knowledge and understanding of CVD risk factors are largely derived from data obtained in developed countries [10] and there is a gap in data from patients with a South Asian background living with RA.

There is wealth of data showing poor outcomes in the non-RA South Asian population with CVD [9, 11, 12]. Several studies [9, 11, 12] have examined barriers to lifestyle change in South Asian populations [13]. Factors such as language barriers, illness beliefs and lack of culturally appropriate opportunities have been cited [3, 14]. Additionally, a review of health promotion in ethnic minority groups emphasized understanding differences of health beliefs [15] and where possible to design cultural sensitive educational programmes for patients of South Asian origin in different countries [15].

While interventions exist for the non-RA population suffering from CVD [16], in rheumatology practice only one study has attempted to address the needs of patients regarding CVD risk [17]. In that UK study targeted White British patients with RA, incorporated cognitive behavioural change strategies [17]. Moreover, that study suggested that education regarding CVD to patients with RA helped them understand that RA is an added risk for developing CVD [17]. However, patients from ethnic minority backgrounds were not included in that study. The current literature in rheumatology highlights the need to develop our understanding around the CVD risk knowledge of patients from South Asian origin living with RA. This is needed to inform clinical practice and inform policy makers of the possibility of a cultural sensitive programme in rheumatology targeting high-CVD-risk groups. Additionally, no study has attempted to compare patient perceptions from two different countries. This would help to develop knowledge around similarities and differences moreover would assist clinicians in planning strategies to reduce long-term cardiovascular morbidity in RA for patients of South Asian origin. With this in mind, the aim of this pilot cross sectional survey was to evaluate the level of knowledge of CVD among patients of South Asian origin with RA. We collected data from India and the UK to see whether there was a substantial difference in the level of knowledge between the two countries that would be worthy of further investigation. In addition, we collected data on patients’ illness perspectives of RA.

## Methods

This cross-sectional study was conducted at the Sanjay Gandhi Institute for Postgraduate Medical Sciences located in the North East of India and the Wolverhampton Royal NHS Trust in the Midlands, UK. Sanjay Gandhi Institute for Postgraduate Medical Sciences is known as one of the centre of excellence for rheumatology with an extensive profile of academic research capturing people from neighbouring regions. The UK centre also is located to capture a large proportion of patients from ethnic minority backgrounds. Consecutive patients with RA were approached during their routine rheumatology appointments in both settings. In India, patients presented from different geographic regions around Lucknow. In the UK, patients presented to New Cross and Cannock Chase Hospitals as part of Royal Wolverhampton NHS Trust which takes referrals from across the Midlands. In India, a member of the research team approached patients while they were waiting to see their consultant. In the UK, patients were approached while waiting to see both consultant or nurse specialist by a member of the research team. If patients expressed an interest in taking part, they were given a Patient Information Sheet outlining the study and time to ask questions before giving their consent. The study was approved by the ethics committees of both institutions (Indian Ethics Committee IEC 2018–65-DM-103- and UK West Midlands Research Ethics Committee 18/WM/0344). Patients were recruited if they: (1) Self-defined themselves as being of South Asian origin and in addition for ‘South Asian’ origin three or more grandparents born in India for UK India or Pakistan; (2) Fulfilled classification criteria for RA [18]. A number of questionnaires were used to collect data from patients. These socio-economic variables were collected (age, gender, occupation and ethnicity) along with clinical data including the disease activity score (DAS) [18]. DAS28 data were extracted from routine follow up clinics around the time when patients took part in the study (within 1–2 months), knowledge about CVD was collected by using the Heart Disease Fact Questionnaire-Rheumatoid Arthritis (HDFQ-RA) [19], a validated self-completion questionnaire [19]. We used both HDFQ-RA 1 and HDFQ-RA 2 containing 26 items to provide a more comprehensive perspective of CVD risk knowledge in patients of South Asian origin. The Illness perception questionnaire (IPQ) was available in Hindi [20, 21].

To accommodate non-English speaking patients in India, HDFQ-RA was translated into Hindi (common language spoken by patients at Sanjay Gandhi) using established guidelines [22]. The validated questionnaire available in English was translated into Hindi by two established rheumatology health experts (SA & AA). First, the questionnaire was translated in Hindi by SA and then corrected by AA for ease of understanding and was adapted to the local population. The following modifications were made: (1) question 3; option living with a partner was removed since this could have appeared to be offensive for the population in India, (2) question 4; options were changed to reflect equivalent school and educational attainments, (3) question 14; many of the options had to be removed or modified as western model of care service do not feature in India. For example, CVD screening clinic, attendance to nurse specialist clinic. The translated HDFQ-RA was also read by a departmental staff member for further independent validity. The aim of this process was to ensure that patients understood the questionnaire. After this exercise, a pilot was conducted with five patients to validate and to ensure that the questions were easily understood. For the UK patients, in whom English was not their first language a hospital translator was used to assist patients to complete the HDFQ-RA. The responses given by patients who filled in the questionnaires themselves and by patients who completed the Hindi HDFQ-RA were checked for reliability of the responses by the statistician (PN). Reliability was adequate for both English (Cronbach’s alpha = 0.81 for HDFQ-RA 1 and 0.82 for HDFQ-RA 2) and non-English versions (0.76 and 0.81).

### Statistical analysis

Data were analysed using SPSS Statistics for Windows Version 22.0 (IBM Corp., Armonk, NY). Categorical data are summarised as counts and percentages. The *t*-test, Fisher’s exact test and Kendall’s tau-b were used to compare the demographic and clinical data. As the disease duration was positively skewed it was log transformed prior to analysis. Patients’ responses to HDFQ-RA 1 and HDFQ-RA 2 were presented as percentages. The general and RA related CVD knowledge scores for each patient were calculated as the percentages of the relevant HDFQ-RA 1 and HDFQ-RA 2 questions answered correctly by each patient. A single general score and a single RA related score were obtained as the means of these scores for HDFQ-RA1 and HDFQ-RA 2. Kendall’s tau-b was used to compare scores between groups. The Mann-Whitney test and Fisher’s exact test were used to analyse the illness perception questionnaire data. A sample size of 118 per group was chosen so that for each question in the HDFQ-RA a two-sided Fisher’s exact test had more than 80% power at the 5% significance level to detect a difference of 20 between the proportions in the two groups expressed as percentages.

## Results

In India, 124 patients were approached for the study. Out of these six declined to participate, three of them due to lack of time and the others because of illiteracy. In the UK, we approached 132 patients of those 14 declined to complete the questionnaires. The main reasons were lack of time and commitment after their clinical appointments.

In total 236 patients (118 from each country) were recruited to the study. The majority of the participants were females (Table 1). In the UK centre, seven participants required assistance in completing the questionnaires via the hospital translator. The level of education as well as employment status was similar in the patients from the two countries. There were more patients in full time employment in India than UK although this difference was not significant. History of co-morbidities such as heart attacks, angina, diabetes, high blood pressure, high cholesterol, obesity was similar in two countries. However, there was a trend towards higher frequency of diabetes (11.9% versus 7.1%), hypertension (28% Vs 25%), and high cholesterol (16.1% Vs 8.6%) among UK patients. Higher numbers of the UK patients were taking cholesterol lowering drugs (*p* = 0.045) and anti-hypertensives (*p* = 0.034).

**Table 1:** characteristic of patients

	Indian	UK	***P*** value
Number	118	118	
Age (years); mean (SD)	44 (12)	45 (12)	0.545**a**
**Female**	83.9%	84.7%	1.000**b**
**Highest level of education**			0.870**c**
1. None	11.0%	5.9%	
2. GCSE/0 level/10th grade	17.8%	26.3%	
3. A-level diploma vocational qualification/12th grade	18.6%	21.2%	
4. Degree	20.3%	12.7%	
5. post graduate	32.2%	33.9%	
**Employment**			0.971**b**
Full time	18.6%	16.9%	
Part time	5.9%	8.5%	
Unemployment	3.4%	2.5%	
Not working due to RA	0.8%	0.0%	
Retired	5.9%	5.9%	
Home maker	61.0%	61.0%	
Student	4.2%	5.1%	
**Age at diagnosis** (years); mean (SD)	37 (11)	38 (11)	0.532**a**
**Height** (metres); mean (SD)	1.56 (0.08)	1.57 (0.09)	0.445**a**
Weight (kilograms); mean (SD)	57.9 (10.2)	57.4 (9.6)	0.675**a**
**History of co-morbidities**			
Heart attack	1.7%	0.8%	0.619**b**
Angina	1.9%	5.9%	0.179**b**
Diabetes	7.1%	11.9%	0.265**b**
High blood pressure	25.0%	28.0%	0.658**b**
High cholesterol	8.6%	16.1%	0.107**b**
Obesity	19.5%	13.6%	0.287**b**
Smoking	2.5%	8.5%	0.083**b**
Previous smoking status	6.8%	7.6%	1.000**b**
Family history of CVD	22.9%	20.3%	0.752**b**
Information on TV or media	36.4%	33.1%	0.682**b**
**CVD and diabetes medications**			
Cholesterol lowering medications	5.5%	13.6%	0.045b *
Blood pressure lowering medications	14.2%	25.4%	0.034b *
Blood pressure of above patients			
Normal	13%	50%	0.017c *
Elevated	6%	0%	
Stage 1	44%	43%	
Stage 2	25%	7%	
Not available	13%	0%	
Insulin	4.5%	2.5%	0.490**b**
Anti-inflammatory drugs	60.6%	53.8%	0.347**b**
Corticosteroids	35.4%	25.4%	0.134**b**
Disease-modifying antirheumatic drugs	100%	100%	1.000**b**
Biological drugs	0%	12.7%	< 0.001b *
DAS 28 mean;(SD)	3.1(0.7)	4.0(0.8)	< 0.001a *
Disease duration (years); geometric mean	5.5	4.1	0.012a *

* = significant at < 0.05 a = t test (for disease duration data were log transformed), b = Fisher’s exact test, c = Kendall’s tau-b

### Disease related variables

The disease activity score was higher in the UK patients than the Indian South Asian patients (4.0 + 0.8 versus 3.1 + 0.7; *P* < 0.001) reporting more tender joints. The disease duration was higher in the Indian South Asian patients (5.5 years versus 4.1 years; *P* = 0.012).

### CVD risk knowledge in general between India and UK

In both countries, patients had poor knowledge of risk factors associated with CVD with only 51.2% patients in India and 51.3% patients in UK getting it correct (Table 2). About half the patients in both countries understood that keeping high blood pressure under control could prevent heart attacks. About 40–50% of patients understood that high cholesterol as well as bad cholesterol could contribute towards developing heart disease. Only 20% of patients knew about role of good cholesterol role in minimising heart attack. About half of the patients were aware that unhealthy diets could cause high cholesterol. Regarding the link between diabetes and heart disease only 47.5% in India and 44.1% in UK knew this. However, patients in both countries understood the CVD risk associated with smoking and the role of quitting smoking. In addition, there were also well aware about the role of exercise and walking in reducing CVD.

**Table 2:** CVD risk knowledge in general between India and UK (*n* = 236)

	India	UK	***P*** value
	% getting it correct	% getting it correct	
Overall general CVD risk knowledge score who got it correct for HDFQ-RA1	44.6%	45.4%	0.734 a
			***P*** **value b**
A person always knows when they have heart disease	26.3%	33.9%	0.256
A person who smokes is more likely to develop heart disease	70.3%	68.6%	0.888
Keeping blood pressure under control will reduce a person’s chance of developing heart disease	56.8%	51.7%	0.514
A person with high cholesterol is more likely to develop heart disease	55.1%	55.9%	1.000
If your ‘good’ cholesterol (HDL) is high you are more likely to develop heart disease	16.1%	24.6%	0.145
Only exercising in a gym or in an exercise class will lower a person’s chance of developing heart disease	34.7%	29.7%	0.486
Eating fatty foods does not affect blood cholesterol levels	50.0%	55.1%	0.515
A person with diabetes is more likely to develop heart disease	47.5%	44.1%	0.695
Overall general CVD risk knowledge score who got it correct for HDFQ-RA2	57.7%	57.2%	0.886 a
			***P*** **value b**
If you have a family history of heart disease, you are more likely to develop heart disease	28.0%	22.9%	0.455
Regular exercise will lower a person’s chance of getting heart disease	80.5%	77.1%	0.633
A person who stops smoking will lower their chance of developing heart disease	70.3%	68.6%	0.888
A person with high blood pressure is more likely to develop heart disease	62.7%	59.3%	0.689
If your ‘bad’ cholesterol (LDL) is high you are more likely to develop heart disease	40.7%	40.7%	1.000
Being overweight increases a person’s chance of developing heart disease	73.7%	78.0%	0.543
The older a person is, the more likely they are to develop heart disease	39.0%	44.9%	0.429
Walking and gardening are considered exercise that will help lower a person’s chance of developing heart disease	66.9%	66.1%	1.000

a = Kendall’s tau-b, b = Fisher’s exact test

### Comparison of RA and CVD risk knowledge between India and UK

Knowledge regarding RA and CVD was even more limited (32.8% in India and 34.4% in UK). Almost 40% of the patients with RA were aware that keeping weight under control, stopping smoking, keeping blood pressure and cholesterol under control can reduce the risk of CVD in patients with RA (Table 3). However, only 15% new that RA increased risk of developing heart disease and a quarter of patients knew about increased CVD risk with anti-inflammatory drugs. Regarding the impact of disease flares over CVD, the knowledge was poor with only a quarter knowing about it. About 20% were aware that steroids increase the risk of diabetes.

**Table 3:** RA and CVD risk knowledge between India and UK (*n* = 236)

	India	UK	***P*** value
	% getting it correct	% getting it correct	
Overall RA related CVD risk knowledge score who got it correct for HDFQ-RA-1	38.1%	40.7%	0.618 a
			***P*** **value b**
A person with rheumatoid arthritis can reduce their chance of heart disease by keeping their weight under control	44.1%	51.7%	0.297
A person with rheumatoid arthritis can reduce their chance of heart disease by stopping smoking	41.5%	49.2%	0.296
People with rheumatoid arthritis should not exercise because it can damage their joints	51.7%	50.0%	0.896
Anti-inflammatory medications, such as diclofenac or ibuprofen, taken by patients with rheumatoid arthritis may increase the chance of heart disease	27.1%	27.1%	1.000
Having lots of inflammation (‘flares’) of rheumatoid arthritis adds to the increased chance of heart disease	26.3%	25.4%	1.000
Overall RA related CVD risk knowledge score who got it correct for HDFQ-RA-2	27.5%	28.0%	0.912 a
			***P*** **value b**
Patients with rheumatoid arthritis are more likely to develop heart disease	16.1%	13.6%	0.715
Rheumatoid arthritis affects the balance of ‘good’ and ‘bad’ cholesterol in the blood in an undesirable way	19.5%	22.0%	0.748
Long term or high doses of steroid medication taken by a person with rheumatoid arthritis may cause diabetes.	15.3%	20.3%	0.395
A person with rheumatoid arthritis can reduce their chance of heart disease by keeping their cholesterol under control	43.2%	39.8%	0.692
A person with rheumatoid arthritis can reduce their chance of heart disease by keeping their blood pressure under control	43.2%	44.1%	1.000

a = Kendall’s tau-b, b **=** Fisher’s exact test

### Comparison of CVD risk knowledge between patients with and without CVDs

A higher proportion of those with existing CVDs knew that a person with high cholesterol is more likely to develop heart disease (77% vs 49%, *p* < 0.001) and that patients with rheumatoid arthritis are more likely to develop heart disease (35% vs 9%, *p* < 0.001). These were the only significant differences.

### Illness perception questionnaire

The IPQ domains of consequences, timeline, and personal control over RA, treatment control, identity, concerns, coherence and emotional representation were similar between patients in the two countries (Table 4). However, patients’ belief about the cause of RA was significantly different between patients from the two countries. UK patients ranked diet (*p* = 0.006) over work (*p* = 0.007) and infection (*p* = 0.036) higher than Indian patients whereas there was no difference in role of genetics and stress. (Table 5).

**Table 4:** Illness Perception Questionnaire

	India	UK	
	Median and inquartile range		*P* value a
Consequences	7 (5–10)	7 (5–10)	0.925
Timeline	9 (5–10)	9 (5–10)	0.838
Personal control	7 (5–9)	7 (4–9)	0.588
Treatment control	10 (8–10)	9 (7–10)	0.301
Identity	7 (5–10)	7 (5–10)	0.767
Concerned	10 (8–10)	10 (8–10)	0.766
Coherence	8 (5–10)	7 (5–10)	0.839
Emotional representation	8 (5–10)	8 (5–10)	0.804

a Mann-Whitney

**Table 5:** Patients’ belief of cause of RA (giving it first rank)

	India	UK	*P* value a
	Number of patients	Number of patients	
Diet	10	26	0.006*
Genetics	7	6	1.000
Obesity	8	9	1.000
Stress	6	12	0.219
Over work	0	8	0.007*
Infection	1	8	0.036*

* = significant at < 0.05 a Fisher’s exact test

### Post hoc power calculation

Based on the observed variability in this study, the sample size of 118 per group had 80% power at the 5% significance level to detect a difference of 10 for general CVD risk knowledge and 12 for RA specific CVD risk knowledge.

## Discussion

This study evaluated the level of CVD risk knowledge among patients of South Asian origin with RA in two different geographical regions. The purpose was to highlight the differences and similarities between two different rheumatology settings (of well-resourced and resource constrained) to identify gaps in our understanding of CVD risk knowledge in patients of South Asian origin with RA. The present study highlights three points: Patients of South Asian origin have limited knowledge of CVD related issues in general. Further knowledge about RA related issues impacting CVD risk was poor and finally no differences were observed between the level of CVD knowledge among patients from the two countries.

In this study, the percentage of participants suffering with diabetes, hypertension, high cholesterol was marginally higher in the UK population as compared to India. Additionally, UK patients were noted to be taking more cholesterol and blood pressure medications. This could be related to better screening and better access to primary health care in UK. Despite the fact that they were on medication, their knowledge was still similar to that of Indian patients. This could be a reflection on clinicians’ inability to communicate key messages to patients.

Interestingly, a number of studies published in the UK show higher rates and earlier onset of CVD due to various predisposition and lifestyle factors in ethnic minorities [9, 11, 23]. Abnormal lipid and especially low-density lipoprotein (LDL) hold a key role in the atherosclerotic processes which has been reported to result in premature atherosclerosis [8]. This has been shown to be elevated in patients of South Asian origin with RA [8]. Additionally, stress plays an important role in developing the CVD risk [24]. In this regard, Solomon and colleagues observed an association between symptoms of tension and carotid plaque occurrence in White and Black Africans RA patients [25]. Furthermore, data suggests that poor adherence to chronic medications may further increase the risk [25]. The ability to fully engage rests on patients’ perceived seriousness of the disease. We have previously shown in the UK this to be linked with patients’ illness and cultural beliefs [26]. Furthermore, patients’ perceived views on causation dedicate their ability to fully control their symptoms [26]. In our study, we found patients’ beliefs about the cause of RA ranking were significantly different between the patients from two countries. People in the UK believed diet, overworking and infection as a cause of RA. Psychological frameworks show that some patients deter from following treatment advice sometimes these are strongly influenced by their ‘common-sense’ beliefs about their illness and treatment [27] indeed we have shown these links in our previous work [26]. Therefore, UK patients may not exercise as they think it will worsen RA. A recent review highlights that stress, lack of exercise and high fat diet were considered the major causes of CVD by South Asians [28].

In India, which has a much higher CVD related death rate of 272 per 100, 000 as compared to global average of 235 per 100,000, the knowledge regarding CVD is low [29]. CVD disease in India occurs at a much younger age and has higher mortality probably related to lack of knowledge leading to late recognition of symptoms and suboptimal health care facilities [29]. In a recent study investigating the acute myocardial infarction (AMI) and sudden cardiac arrest in Beijing, Shanghai and Bangalore, a baseline assessment of public knowledge, attitudes and practices was performed [30]. That study reported that the CVD risk knowledge was only modest. Smoking was identified as risk factor by over 90% while hypertension and high cholesterol was named as risk factor by only half and less than quarter knew that diabetes and lack of exercise increase the risk of CVD. This has also been found in our study. Almost one quarter could not name any symptom which suggested a myocardial infarction while only 60% recognized chest pain as an important symptom [28]. That survey also suggested that the physicians were not counselling patients on the CVD risk factors or its symptoms. Moreover, the co-morbidities were not reported in that study.

Similar studies across the globe also show the knowledge gap between the Caucasians and the South Asian patients as regards CVD risk knowledge [14, 16]. Studies on patients with CVD and diabetes all highlight the fact that patients of South Asian origin have significant barriers to acquiring sufficient knowledge and therefore have low level of knowledge [9, 11]. Patients of South Asian origin have been found to be concerns about the long-term management of their underlying illness [11]. Studies further report associations with lack of knowledge about the long-term consequences of the illness and cultural stigma attached to chronic illness [31, 32].

There are only a few studies that assess CVD knowledge in patients with RA. John et al. [19] reported that in UK Caucasians with RA nearly 80% of respondents were aware of the modifiable risk factors for CVD, in contrast to only 50% in our study. Similar to our data, patients in the John et al. study were reported to have poor knowledge about increase in CVD risk related to anti-inflammatory drugs and RA flare. This suggests that rheumatology clinics are not equipped to educate the patients about heart disease. Moreover in a study of South Korean patients reported that RA patients perceive their CVD risk to be less as compared to that calculated using the Framingham risk score. This also suggests a knowledge gap [33].

Our results pose a challenge and an opportunity in a very important clinical arena – that is, how to increase the CVD risk knowledge in patients of South Asian origin. The UK health services offer more opportunities to screen the CVD risk and educate patients and implement change. These range from general lifestyle, diet, physical and exercise [16]. Behavioural change and cultural influences have also been explored extensively [15]. Interventions such as Khush Dil [16] have been employed to reduce the risk of CVD in patients of South Asian origin. We strongly recommend similar educational programme in the rheumatology clinics are required that includes behavioural changes towards exercise, quitting smoking, reducing weight, adherence to all medication including lipid lowering drugs, anti-hypertensives and DMARDs. In addition, we also need to communicate with patients about the effective control of RA disease activity and long-term consequences of flares in RA and its added risk to developing CVD. Recently in the UK, leaflets as well as cognitive behavioural programs have been developed in English to increase an awareness of CVD risk knowledge among patients with RA [10, 17]. These are implemented in collaboration with National Rheumatoid Arthritis Society (NRAS). For patients of South origin with lower literacy level Apni Jung (our fight against rheumatism) (www.nras.org.uk/apnijung) has been developed as a platform on the NRAS website. We need to develop programmes in CVD risk knowledge that are culturally sensitive on the existing platform so that these can be accessed both in the UK and India.

The study had some limitations. Data on Framingham Risk Score in these patients from both countries would have helped us to know the actual risk of CVD and then we could have studied the relationship between knowledge about CVD and risk in an individual. Data on patients’ understanding of RA could have provided insights into their knowledge and whether those with poor understanding of RA also had poor CVD risk knowledge. Data on medication therapy for RA would have helped us to know the possible cause of higher disease activity in the UK patients. Translating questionnaires into different languages is always a challenge we recognise this as a limitation. In future study the HDFQ-RA 1 and HDFQ-RA 2 will require a larger group of patients for validation. This pilot study demonstrates that the use of HDFQ-RA 1 and HDFQ-RA 2 can be useful to understand the CVD risk knowledge in patients of South Asian origin living with RA. Due to funding restrictions, data were collected only from two centres. Despite these limitations, for the first time, our study suggests that people of South Asian origin have poor CVD risk knowledge regardless of whether they are in resource rich or resource poor settings. Further larger scale study is required to confirm this.

## Conclusion

This novel study highlights CVD risk knowledge in two different countries. Despite many awareness campaigns in the UK patients still displayed similar “general” CVD risk knowledge to patients in India. Poor RA specific CVD risk knowledge suggests a need to educate ethnic minorities in developed world as well as all patients in developing country like India.

## Data Availability

All data generated or analsyed during this study are included in this published article [and its supplementary information files] or are available from the corresponding author on reasonable request.

## Abbreviations

RA: Rheumatoid arthritis
CVD: Cardiovascular disease
HDFQ-RA: Heart Disease Fact Questionnaire-Rheumatoid Arthritis
